# Supplementary material for: Sacbrood viruses and select Lake Sinai virus variants dominated Apis mellifera colonies symptomatic for European foulbrood
Source: Microbiol Spectr. 2024 Jul 9;12(8):e00656-24. doi: 10.1128/spectrum.00656-24 (PMC11302354; doi:10.1128/spectrum.00656-24)
Supplement: Supplementary material — Table S1; Fig. S1 and S2. [file spectrum.00656-24-s0003.docx]

**Supplemental Table 1.** Results of ADONIS test. Significant p-values are in bold.

| **Interaction** | **Df** | **SumsOfSqs** | **MeanSqs** | **F.Model** | **R2** | **Pr(>F)** |
| --- | --- | --- | --- | --- | --- | --- |
| EFB | 1 | 0.592 | 0.591 | 2.438 | 0.008 | 0.056 |
| Yard | 1 | 0.274 | 0.273 | 1.126 | 0.003 | 0.302 |
| Virus species | 4 | 3.602 | 0.900 | 0.0488 | 0.048 | **0.001** |
| EFB:Yard | 1 | 0.391 | 0.391 | 1.610 | 0.005 | 0.155 |
| EFB:Virus species | 4 | 3.08 | 0.769 | 3.171 | 0.041 | **0.001** |
| Yard:Virus species | 4 | 2.27 | 0.568 | 2.342 | 0.030 | **0.007** |
| EFB:Yard:Virus species | 4 | 0.944 | 0.236 | 0.97 | 0.012 | 0.465 |
| Residuals | 8 | 62.632 | 0.242 |  | 0.848 |  |
| Total | 11 | 73.788 |  |  | 1.000 |  |

**
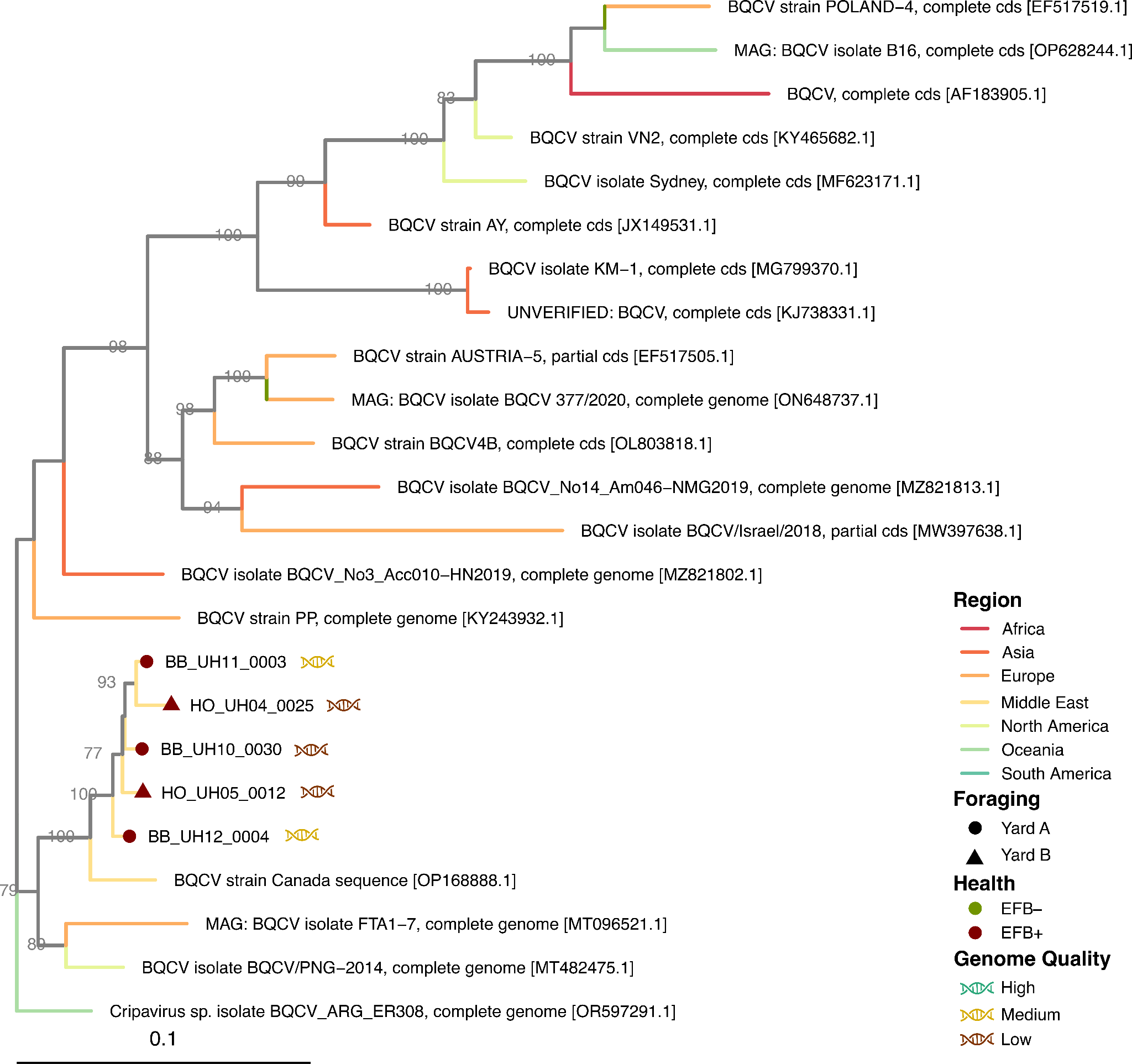
**

**Supplementary Fig 1.** Whole-genome maximum-likelihood phylogeny of viral genomes from EFB symptomatic and asymptomatic bee viromes for black queen cell virus. Branches are coloured by the origin of viral genomes (see Supplementary Data file 2 for full metadata of genome used in phylogenies). Branch support values are from left to right, bootstrap from 1000 replicates reported as a proportion out of 100. Viral genomes accessed from NCBI are denoted by their accession numbers, while genomes assembled in this study are annotated with their EFB health record, their holding yard, and genomes by their quality.

**
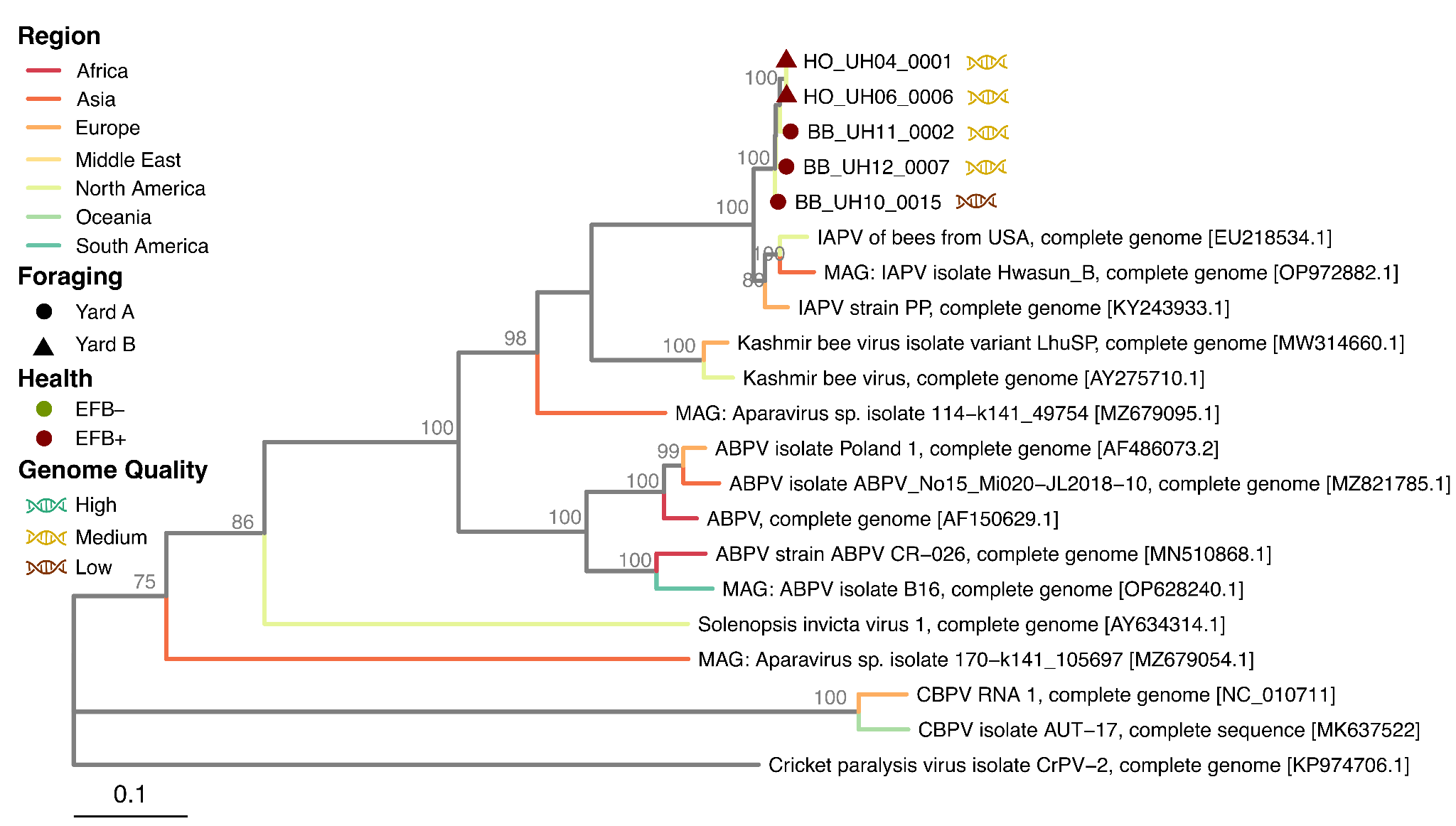
Supplementary Fig 2.** Whole-genome maximum-likelihood phylogeny of viral genomes from EFB symptomatic and asymptomatic bee viromes for AKI complex viruses. Branches are coloured by the origin of viral genomes (see Supplementary Data file 2 for full metadata of genome used in phylogenies). Branch support values are from left to right, bootstrap from 1000 replicates reported as a proportion out of 100. Viral genomes accessed from NCBI are denoted by their accession numbers, while genomes assembled in this study are annotated with their EFB health record and foraging treatment, and genomes by their quality.
